# Supplementary material for: Cognitive-behavioral correlates of pupil control ideology
Source: PLoS One. 2021 Feb 10;16(2):e0246787. doi: 10.1371/journal.pone.0246787 (PMC7875416; doi:10.1371/journal.pone.0246787)
Supplement: Attachment — Submitted filename: Response to reviewers.docx [file pone.0246787.s001.docx]

Dear Dr. Natasha McDonald,

Thank you for the opportunity to revise the manuscript entitled “Cognitive-behavioral correlates of pupil control ideology” (PONE-D-19-32116).

We addressed all concerns raised by the reviewers. The manuscript's revised form contains significant changes in the introductory part to address two important questions raised by the reviewer 1 (Why focusing on pupil-control ideology? and Why does it matter to investigate the association between the pupil control ideology and many, apparently, overlapping beliefs?) We also improved our analysis section. We complemented our zero-order correlation analyses with partial correlation analyses by investigating whether the target relationships remain significant when controlling for several teaching-related demographic variables. We also addressed the family-wise error concern by dividing our analyses into primary and secondary (post-hoc / exploratory) analyses and selecting a more conservative p-value for the exploratory analyses.

In the following lines, you can find the way we addressed each point raised by the reviewers. The text written with a black font represents the reviewers’ comments. The text written with the blue font represents our answer to these suggestions.

***

Reviewer #1: Based on a sample with N = 155 teachers, this study investigated correlations between teachers’ pupil-control ideology and different types of beliefs that are suggested to be relevant in cognitive-behavioral therapy. Results of correlation analyses revealed that teachers with control ideology had more dysfunctional beliefs than the ones with a humanistic ideology.

I appreciate the general idea guiding this paper and find merit in investigating how teachers’ beliefs shape their relationships with students. However, I was missing a compelling theoretical rationale and empirical evidence supporting your idea to focus on pupil-control ideology. Because no data on how teacher beliefs play out in the classroom (or perhaps for teacher well-being) is presented, I am concerned that this contribution does little to answer the question of how we can help teachers improve the quality of their relationships with students (in discussion).

The initial manuscript contained a couple of references to provide empirical support for the link between the pupil control ideology and well-being related concepts such as burnout, stress, and self-efficacy. In the revised version we extended such evidence, and we included more arguments for the relevance of this control ideology for the quality of the teacher-student relationship.

In addition, this cross-sectional study only relies on teacher self-reports of various types of profession-specific (i.e., pupil-control ideology) and general beliefs (i.e., dysfunctional beliefs derived from cognitive-behavioral therapy) and merely investigates correlations. The pending question here is: Why does it matter? Why do we need to know how pupil-control ideology correlates with the many beliefs that seem to measure conceptually overlapping constructs? In addition to these general concerns, I will provide some more detailed suggestions below.

Thank you for pointing out a possible view from readers specialized in educational sciences who are less familiar with cognitive therapy concepts (why do we need to know how PCI correlates with what it seems, many overlapping beliefs constructs).

We added two paragraphs in the introductory part to address this point, which we hope will bring more clarity to what dysfunctional beliefs are. We also stressed that the objective of any cognitive-behavioral intervention (e.g., to reduce the level of teachers' stress) is to identify and alter dysfunctional beliefs.

We acknowledged the cross-sectional approach's limitation in the discussion section of the initial version of the manuscript. We continue to do so, given that correlation does not necessarily imply causation. However, we also want to highlight the strength of the current study. It is the first time when even a correlational investigation of the link between the pupil control ideology and dysfunctional beliefs is enacted. The expected association between the more specific (pupil control ideology) and more general (e.g., irrational beliefs, schemas) might provide clues on the (more general) cognitive vulnerabilities of teachers that are addressed via cognitive-behavioral interventions. In such cases, the benefits are not limited to the well-being of teachers, as the pupil control ideology is also highly relevant for the quality of teacher-student interaction.

Most of the above concerns were addressed in the introductory part of the paper. We hope that these revisions will contribute to a better rationale for the explicit link between PCI and dysfunctional beliefs. The study represents a first step in opening a doorway for cognitive interventions that could change a teacher-student relationship's quality.

Abstract

I am not sure that pupil control ideology is a well-known term so you may have to clarify its meaning in the abstract already. Likewise, I imagine it will be difficult to know for readers with a background in education what “personal beliefs originated from cognitive-behavioral therapy” are.

Thank you for pointing this out. We added a description of the pupil control ideology in the abstract. Likewise, we referred to dysfunctional beliefs as a specific term coined in cognitive-behavioral therapy literature to reduce the ambiguity of some abstract statements.

Introduction

In general, I think the manuscript would profit from more precise definitions of key terms.
You claim that teachers’ pupil control ideology is a “core construct for the quality of teacher-pupil relationship”, but where is the evidence supporting this strong claim? In my view, you need to be much more specific in terms of the (empirical) relevance of pupil control ideology for teacher-student relationships, teacher well-being or other key outcomes in the teaching profession. If you cannot support the relevance of pupil control ideology, I am quite skeptical that educational research and practice could profit from the insights your study provides. You also may want to provide more detail in this regard.
Given the central role of teacher-student relationships in your manuscript, I would like to see a clear definition. On p. 4 you cite a few studies which are supposed to underpin the relevance of pupil control ideology for the teacher-student relationship, but I am not convinced that things like corporal punishment, teaching efficacy, and academic optimism are the same as a high-quality teacher-student relationship.

We introduced such definitions, and we provided more theoretical and empirical arguments to support our "claim" that the pupil control ideology (the way teachers control students' classroom behavior) is relevant for setting the tone for the teacher-student relationship (interaction). We did not claim that academic optimism, or the attitude towards corporal punishment, are the same as a high-quality teacher-student relationship. Still, such aspects are relevant for the teacher-student relationship (e.g., if, by instance, a teacher who has a positive view on corporal punishment uses such disciplinary methods to control their students).

A more detailed definition of automatic thoughts, schemas, and irrational beliefs would be helpful. Moreover, I was wondering how pupil control ideology and the beliefs from cognitive-behavioral therapy are actually distinct from one another. That is, could you also view pupil control ideology as a profession-specific type of these beliefs? Due to the strong theoretical overlap between all of the constructs you are measuring, I imagine that factor analyses are necessary to get a clearer impression of how the things you measure are distinct from one another or rather very similar.

As already pointed, we addressed this issue in the revised version of the manuscript (e.g., see a brief overview of the dysfunctional beliefs section and the first paragraph of the present study). We also provide empirical correlations for our main research questions (how pupil control ideology relates to various dysfunctional beliefs) and found significant support for the lack of overlap between these constructs, based on the coefficient correlations' small magnitude. The potential remaining overlap between different layers of dysfunctional cognitions is already recognized from a theoretical perspective. Changes in upper-level layers (e.g., core beliefs) most probably is likely to determine subsequent changes in the other layers, whereas empirical "confirmation" of beliefs in a specific context reinforces/stimulate the emergence of more general, cross-situational beliefs.

Overall, I found the presentation of hypotheses not easy to follow. For instance, I did not understand whether “unconditional self-acceptance” is a superordinate concept for demandingness beliefs, frustration tolerance beliefs, etc. or whether these terms are independent of each other. Furthermore, I found it confusing that perfectionisms was mentioned in the first few hypotheses and then again at the very end of the introduction.

We hope the new section on dysfunctional beliefs that explicitly states the existence of three layers of beliefs within the cognitive-behavioral framework is now also helpful for understanding the presentation order for hypothesizes. For instance, the mention of perfectionism in the first few hypotheses was a hypothesis that focuses on automatic thoughts (with perfectionistic content). This is the first layer of cognitions in the psychopathological model.

Method and Results

Because you analyze the multiple dimensions of the perfectionism scale separately, you should mention each subscale in the description of the instruments and report Cronbach’s alpha for each of them.

Thank you for pointing out this inconsistency. We added the requested info

Why were different scales used for measuring the same construct (e.g., ABS, SPB, and YSQ, two perfectionism scales)?

They do not measure the same construct, but different related cognitions, as we now made more explicitly through a detailed definition of the CBT-related constructs. Thus, YSQ refers to schemas (core beliefs). In contrast, ABS and SPQ refer to intermediate beliefs, but without covering the same domain (e.g., ABS-2 includes only self-directed should, whereas SPQ has the merit to disentangle self-directed should from other-directed should). These differences are usually well-known in the CBT field.

As mentioned earlier, I think factor analyses would be necessary to investigate whether the various different scales you included are empirically distinct. A correlation table for all included measures would add to this.

We reiterate that the potential overlap between different dysfunctional cognitions layers is theoretically assumed and meaningful from a clinical perspective. Besides, it has already been addressed in previous studies. Interested readers could refer to the empirical work of Sava (2009) or Calvete, Orue, & Hankin, 2013), or to classical cognitive therapy manuals such as Leahy (2004) or Dobson (2012).

There is a large number of statistical tests. Thus, the authors may want to consider adjusting alpha to avoid false positive findings.

Based on Reviewer 1 and Reviewer 3 suggestions, we introduced a data analysis strategy section in the method section to address these concerns. For instance, we divided the results as primary findings (related to our hypothesizes) and secondary findings (other exploratory / less relevant associations) to address the family-wise error issue.

In Table 1 it is difficult to evaluate whether the mean values are in a high or low range.

We included both the potential range and the actual range of results obtained in our sample.

Moreover, it is difficult to see at first glance which of the correlations are statistically significant and which are not.

The new table includes now asterisks for the statistically significant coefficients.

Minor points

A reference is missing in line 83/84

Thank you for pointing it out. We included the reference.

All statistical symbols should be printed in italics

We made these corrections.

The reference list should be checked for consistent spelling (e.g., capitalization of first letters)

Thank you for pointing out this aspect. The reference list is now consistent in the way it is spelled.

Reviewer #2: The topic selection of this paper has certain practical significance. The paper provides some innovative results. The literature review is sufficient. The method is reasonable. The discussion was in-depth.

Thank you for your appreciation!

Reviewer #3: Thank you for the opportunity to review, “Cognitive Behavioral Correlates of Pupil Control Ideology” for PLOS ONE. This study examined the relations between pupil control, defined on a spectrum from humanistic to custodial, and teachers’ perceptions of perfectionism and hostile automatic thoughts. The authors found that teachers with more custodial perceptions of control had higher ratings of perfectionism and hostile thoughts, but no relation was found for punitiveness schema. Overall, the manuscript is generally clear and the study provides some interesting insights into the relations of the constructs. That being said, there are few concerns. First, it is unclear if the hostile thoughts measure was adapted to refer to hostile thoughts towards students. Although general thoughts of hostility towards others are potentially correlated with thoughts about students, there is a difference and that should be made explicit.

Thank you for pointing out this lack of clarity! We made it explicit that all CBT-related measures have been collected using their standard instruction, referring to their thinking style in general, not only in the school context. It will also ensure consistency in the way we measured all cognitions.

Second, a brief data analysis section in the method section should be added. In particular, the authors should discuss using one-tailed tests and, per Table 1, two-tailed test and justify each.

We introduced this data analysis strategy section at the end of the method section. Thank you for this useful suggestion.

Relatedly, the author should consider the likelihood of family-wise error rates given the series of correlations of related constructs. The author also needs to provide information about how they scaled the measure scores and the min/max values. Lastly, the reliance on correlations does not necessarily address the researchers’ primary interests and do not control for potentially relevant covariates, including years teaching. The data provide opportunities for some mediation analyses (e.g., do irrational beliefs mediate the relation between PCI and Hostility) and, more importantly, comparison of groups. The authors could create groups of humanistic and custodial teachers and then use t-tests, or ideally, a regression or ANCOVA model controlling for teacher characteristics.

This chunk of suggestions brings up several issues. For some of them (additional info, controlling for potentially relevant covariates, considering the likelihood of family-wise error) we implemented the suggested steps. For instance, table 1 has been reshaped to include not only zero-order correlations but also partial correlation values controlling for teacher’s gender, years of teaching, the school level (e.g., lower secondary vs. upper secondary), and school area (urban vs. rural). Likewise, we included actual and theoretical score ranges for each variable. Other suggestions (e.g., mediation analysis) were not related to our main research question (which was “is there a link between the pupil control ideology and dysfunctional beliefs?”). Likewise, a comparison of contrast groups (e.g., custodial teachers vs. humanistic teachers) is problematic for several reasons. From a measurement perspective, there are no prior established cut-off scores to differentiate between these two groups. Therefore it would not be easy to say at precisely what score a teacher becomes custodial. Besides, from a theoretical perspective, the two types are extreme poles of a continuum variable, and therefore it would be less appropriate to treat them as discrete variables. Nonetheless, a dichotomization of the continuous variable will also affect the study's statistical power by losing significant power.

Page 6- line 101- there should be “ before My Work

Thank you! We corrected.

Page 8- name the author for reference 28 as the sentence says, “proposed by…”

Thank you! We corrected.

Page 9, line 175- consider “incentive” instead of “reward”

Thank you! We made the suggested word change.

Page 10- reliability coefficients should be reported for the Survey of Personal Beliefs.

The reliability coefficients for the SPB scale are now included.

Page 11- reliability coefficients should be reported for the Youth Schema Questionnaire.

We included the reliability range for the 18 YSQ scales.

We thank all three reviewers for their useful comments and appreciation!
