# Supplementary material for: Cognitive-behavioral correlates of pupil control ideology
Source: PLoS One. 2021 Feb 10;16(2):e0246787. doi: 10.1371/journal.pone.0246787 (PMC7875416; doi:10.1371/journal.pone.0246787)
Supplement: Attachment — Submitted filename: Response to reviewers.docx [file pone.0246787.s002.docx]

Dear Dr. Natasha McDonald,

Thank you for the opportunity to revise the manuscript entitled “Cognitive-behavioral correlates of pupil control ideology” (PONE-D-19-32116R1).

In the following lines, you can find how we addressed each of these three points that remained after the first revision. The text written with a black font represents the reviewers' comments. The text written with the blue font represents our answer to these suggestions.

***

Editor of this manuscript: The authors have done an excellent job revising their manuscript. That being said, I would like the authors to address the first two concerns noted by Reviewer #1, namely a little more justification for the research question and, importantly, noting the reliability concerns in the limitations section.

Reviewer #1: First, I would like to thank the authors for the thorough revision of their manuscript. In particular, I value the added detail on dysfunctional beliefs and inclusion of clearer definitions. Nonetheless, I still find myself unconvinced of the theoretical and practical relevance of the research questions. To make the paper more convincing for the readers, I suggest you explain why these insights are of interest. Simply saying that the correlation between custodial views and irrational beliefs has not been investigated is not really an argument from my perspective.

In the revised version, we extended the study purpose by including one additional page. We explained: (a) why the pupil control ideology is worth being studied from a psychological perspective, and not only from an educational sciences perspective; (b) using the cognitive-behavioral framework adds practical value in addressing this topic. I inserted below the text that was introduced to address the concern of Reviewer 1.

Addressing this research question is relevant for both theoretical and practical reasons. From a theoretical perspective, the study contributes to enriching the literature on pupil control ideology underpinnings. As already underlined, most previous studies have focused on antecedents and consequences of pupil control ideology from an educational science perspective. There were significantly fewer studies that aimed at addressing this topic from a psychological perspective. Teachers strive to achieve a sense of competence and success. However, their efforts are often undermined by the lack of personal control and unpredictability in a classroom context, leading to stress or burnout. Most psychological studies have focused on depicting the custodial teacher personality profile (e.g., more authoritarian, less open, etc.). Such findings could help determine who the teachers at-risk for manifesting a problematic teacher-student interaction are. However, they are less useful for an intervention purpose because personality traits are stable and difficult to change. Such findings are also less relevant to the aim of decreasing the level of stress among teachers.

That is why examining the link between dysfunctional beliefs and the pupil control ideology is of more practical value. The core of any cognitive-behavioral intervention consists of replacing dysfunctional beliefs with more functional ones. Such interventions could provide a new avenue to alter pupil control ideology indirectly by changing the correlated dysfunctional beliefs. Changing teachers' cognitions on aspects such as perfectionistic cognitions or their demandingness beliefs will most likely influence their interaction with students by altering the amount of control they exert in the classroom context. Likewise, addressing teachers' cognitions that could impair interpersonal interaction, such as hostility or entitlement, will change teachers' beliefs about pupils with whom they interact daily, towards perceiving a less threatening classroom environment. Thus, our study opens an avenue towards potential cognitive-behavioral interventions in the school setting by first gathering evidence on the association between the pupil control ideology and specific dysfunctional beliefs. Such interventions have the potential of leading to multiple direct and indirect benefits regarding the classroom climate, classroom management, and teachers and pupils' well-being.

Reviewer #1: Furthermore, I noticed that there were some scales with reliabilities with α < .70 (i.e., some subscales of ABS 2, SPB, Young Schema Questionnaire, and Perfectionism Inventory). This is an issue you may want to address in the discussion.

Thank you for bringing up this issue. We delineated two main limitations of the current study, and we changed the text accordingly in the discussion section. I inserted the relevant paragraph below.

The empirical results from this study should be considered in light of some limitations. First, the cross-sectional design for the present research prevents us from empirically establishing whether such cognitive correlates are indeed antecedents for the pupil control ideology teachers adopt. Likewise, in the absence of a longitudinal design, we cannot analyze these relationships in their dynamics. Some dysfunctional beliefs (e.g., derogation of others) could result from teachers' emotional distress, caused by their perfectionistic and rigid demands. Second, some of the scales used to measure dysfunctional beliefs had low reliability, with Cronbach's alpha lower than .70. These values signal a significant level of measurement error in data, leading to a decrease in the statistical power to address some of the present study hypotheses. However, despite the attenuation of the effect size, most primary research hypothesizes were supported by data.

Reviewer #1: Finally, I still think that factor analyses are necessary and the authors’ argument that an overlap between different dysfunctional cognitions layers is expected underpins this thought rather than dispelling it. Imagine that, for example, ten of the dysfunctional beliefs are correlated by r = .70 and load on the same factor. Then, one would argue that they measure the same underlying construct. Hence, finding ten statistically significant correlations between the ten dysfunctional beliefs and custodial orientations would seem impressive, when in fact you simply investigated ten “variations” of one and the same construct making it little surprising that all of them are associated with custodial orientations in similar ways.

Conducting such an analysis in our study is not recommended, either theoretically or empirically.

From a cognitive-behavioral perspective, there is no rationale to put in the same basket core beliefs, intermediate dysfunctional beliefs, and automatic thoughts as they differ in their stability, situational consistency, level of abstraction, and utility. That is why they are organized hierarchically in different layers of cognition. In the same way, one will not mix items with facets scores and domains scores when conducting factorial analyses in the personality field, for example.

The very high correlation between any two constructs is not sufficient to disregard one of the constructs. For instance, depression and anxiety correlate highly (around .70), and this did not lead to the suggestion of dropping out one of these two constructs. Nor in the case of low self-esteem, despite its high correlation with depression. Therefore, two strongly correlated constructs could still keep their relevance, based on their discriminative validity and theoretical relevance, despite their high correlation. Take for example, the case of the ABS-2 instrument. All scales included in the instrument are highly correlated (in the range of .50 - .70). Despite the high correlation among each other, each scale is good predictor for a specific negative emotion (e.g. the low frustration scale for anger, the self-downing scale for sadness, the awfulizing scale for anxiety). Therefore, from a measurement perspective, all scales used in the current study are taken from the existing literature, they are not new scales. Interested readers could refer to their original validation studies and subsequent methodological studies.

From a data sample perspective. Our sample is based on data gathered from approximately 150 participants. It is improbable to find a stable factorial solution in an analysis that involves about 40 variables (scales). Likewise, very high correlations were extremely uncommon in our sample. Out of 946 pairs of correlations at the scales level, only 7 (<1%) were within the range of .70 - .75, and only 36 (3,8%) were in the range of .60 to .69.

From a factorial analysis perspective, we know that a factorial solution is strongly dependent on the input variables. For instance, addressing the factorial solution of the perfectionism scale at the item-level reveals perfectionism's multidimensional nature. Dropping the item-level and conducting a factorial analysis using only the scale scores as input leads to the extraction of a single factor, the overall perfectionism level. Therefore, the factorial solution is highly dependable on input (variables to be analyzed).

In the absence of a theory-driven or data-driven rationale to conduct such a factorial analysis, such an endeavor is likely to remain just a technical exercise. Therefore, we did not include such an analysis in the manuscript. First of all, because it would address a different purpose than the aim of the current manuscript. Second, because it is not theoretically and methodologically supported to mix automatic thoughts, intermediate beliefs, and core beliefs.

***

To conclude, we would like to thank both Reviewer 1 and Reviewer 2 for their appreciation and suggestions! We hope that the research question is now better justified in terms of its relevance and that the study's limitations are better addressed. Therefore, we hope that the manuscript's second revision has led to an improved version that the previous version of the manuscript.
